# Use It or Lose It? A Meta-Analysis on the Effects of Resistance Training Cessation (Detraining) on Muscle Size in Older Adults

**DOI:** 10.3390/ijerph192114048

**Published:** 2022-10-28

**Authors:** Jozo Grgic

**Affiliations:** Institute for Health and Sport, Victoria University, Melbourne, VIC 3011, Australia; jozo.grgic@live.vu.edu.au

**Keywords:** strength training, elderly, muscle hypertrophy, sarcopenia

## Abstract

This review aimed to explore the effect of resistance training cessation (detraining) on muscle size in older adults. Five databases were searched to find eligible studies. Their methodological quality was assessed using the PEDro checklist. The data were pooled in a random-effects meta-analysis. Six studies, with eight groups, were included in the review. Resistance training interventions lasted from 9 to 24 weeks. The detraining duration was from 12 to 52 weeks. Studies were classified as being of fair or good methodological quality. Compared to the baseline data, muscle size significantly increased following the resistance training intervention (Cohen’s *d*: 0.99; 95% confidence interval: 0.63, 1.36). Compared to the post-resistance training data, there was a significant decrease in muscle size following training cessation (Cohen’s *d*: −0.83; 95% confidence interval: −1.30, −0.36). In subgroup analyses, there was no significant decrease in muscle size following 12–24 weeks of training cessation (Cohen’s *d*: −0.60; 95% confidence interval: −1.21, 0.01). There was a significant decrease in muscle size following 31–52 weeks of training cessation (Cohen’s *d*: −1.11; 95% confidence interval: −1.75, −0.47). In summary, resistance training increases muscle size in older adults. In contrast, training cessation is associated with a decrease in muscle size. However, the loss of muscle size might be related to detraining duration, with greater muscle loss occurring during longer duration detraining periods. Future studies are required to establish the time course of muscle size changes during detraining in older adults.

## 1. Introduction

Older adults are one of the fastest-growing populations in the world [1]. This population is commonly affected by dynapenia and sarcopenia. Dynapenia is defined as the age-associated loss of muscle strength [2]. Sarcopenia can be defined as a progressive state characterized by a degenerative loss of muscle mass and function [3]. Sarcopenia is associated with an increased likelihood of falls, fractures, physical disability, and mortality [3]. There are estimates that the prevalence of sarcopenia in older adults is between 10% and 27% [4]. Resistance training is an exercise form that can prevent and manage sarcopenia in older adults [5]. Studies have demonstrated that resistance training increases muscle size in various populations, including youth, adults, and older adults [6,7].

After 65 years of age and under periods of inactivity, muscle size declines yearly by ∼0.4% to ∼1% [8]. Due to these effects, resistance training is recommended for older adults [9]. While resistance training increases muscle size in older adults, less is currently known about the trajectory of muscle size changes during training cessation (detraining). Training cessation may occur due to various reasons (e.g., because of an injury, loss of motivation, travel). Older adults might also need to cease resistance training at specific periods because of illness. This topic is also timely due to recent events associated with the COVID-19 pandemic, which often limited access to gyms [10]. 

Given that resistance training increases muscle size in older adults, it is generally assumed that a decrease in muscle size will occur when this exercise stimulus is no longer applied (i.e., during detraining) [11,12,13,14,15,16]. Several studies have corroborated this hypothesis. For example, one study [16] reported that 12 weeks of resistance training increased muscle size by 6%, followed by a 5% decline after 24 weeks of detraining. While this study reported a small decrease, other studies have observed a large decline in muscle size (−20%), which occurred during even shorter periods of detraining [11]. As the estimates varied between studies, it is not clear what the magnitude of the decline in muscle size during detraining is, highlighting the need to pool these results to obtain an overall estimate. A detailed examination of the current evidence also highlights that not all studies reported a reduction in muscle size of older adults during detraining. For example, one study utilized a design with 24 weeks of resistance training and 24 weeks of detraining [15]. In contrast to the previously reported data, muscle size was maintained during detraining [15]. Thus, currently available data are conflicting in terms of the magnitude of detraining′s effects (i.e., a small or large decrease in muscle size) and the direction of the effect (i.e., a decrease or no decrease in muscle size).

Given the conflicting evidence and the established importance of skeletal muscle in older adults, the aims of this review were to: (i) examine the effects of resistance training on muscle hypertrophy in older adults; and (ii) explore the effects of detraining on muscle size. 

## 2. Methods

### 2.1. Search Strategy 

The search for this review was performed in two phases. In the first phase, a search through bibliographic databases was performed. Specifically, this phase of the search process was carried out in Academic Search Elite, PubMed/MEDLINE, Scopus, SPORTDiscus, and Web of Science. In all of these databases, the following search syntax was applied: (“detraining” OR “training cessation”) AND (“elderly” OR “older adults” OR “older men” OR “older women” OR “oldest old” OR “oldest-old” OR “very old” OR “advancing age” OR “advancing years” OR “old-old” OR “old old” OR septuagenarian* OR nonagenarian* OR octogenarian* OR centenarian*) AND (“resistance training” OR “resistance exercise” OR “weight lifting” OR “weightlifting” OR “strength exercise” OR “strength training” OR “strengthening” OR “resistive exercise” OR “resistive training”) AND (“muscle hypertrophy” OR “muscular hypertrophy” OR “muscle mass” OR “lean body mass” OR “muscle fiber” OR “muscle size” OR “muscle fibre” OR “muscle thickness” OR “cross-sectional area” OR “cross sectional area” OR “computed tomography” OR “magnetic resonance imaging”). In the second phase of the search process, forward and backward citation tracking was utilized. Forward citation tracking included examining studies that cited the included studies. Backward citation tracking included examining the reference list of the included studies. The search for studies was completed on 1 March 2022. 

### 2.2. Inclusion Criteria 

Using the PICO criteria, this review included the following studies: population (P)—older adults aged ≥65 years; intervention (I)—resistance training followed by a period of training cessation (detraining); comparison (C)—pre-intervention, post-intervention, and post-detraining data; outcome (O)—muscle size evaluated using ultrasound, magnetic resonance imaging, computed tomography, or muscle biopsies [17].

### 2.3. Data Extraction

Data extraction from the included studies was performed for the following variables: lead author name and year of study publication; participants’ characteristics (e.g., sex, age); details on the resistance training intervention; detraining duration; site and tool used for muscle size evaluation; baseline, post-intervention, and post-detraining muscle size mean ± standard deviation data.

### 2.4. Methodological Quality

The quality of the included studies was appraised using the PEDro checklist. The PEDro checklist has 11 items that evaluate various methodological aspects [18]. These include randomization, blinding, allocation concealment, data reporting, attrition, and inclusion criteria. The answers to all items on the checklist are binary (“yes” or “no”), where only the “yes” answer is associated with a point. The first item does not contribute to the summary score and therefore, the maximum number of points on the checklist is 10. Based on the summary scores, studies were classified as poor, fair, good, or excellent quality if they scored ≤3 points, 4–5 points, 6–8 points, and 9–10 points, respectively [19].

### 2.5. Statistical Analysis

All meta-analyses were performed using standardized mean differences (Cohen’s *d*). The mean ± standard deviation muscle size data and the data on the number of participants were used to calculate standardized mean differences. The effectiveness of the training programs in increasing muscle size was first examined by comparing the pre-intervention vs. post-intervention data. Next, muscle size data collected post-intervention vs. post-detraining were compared to explore the effects of training cessation. Finally, subgroup meta-analyses were performed to examine the effects of detraining duration (12–24 weeks vs. 31–52 weeks). In the two main analyses, additional sensitivity analysis was performed by excluding the data from one study [14] that included two groups, one that received recombinant human growth hormone and one that received a placebo. The interpretation of effect sizes was based on the following thresholds: trivial (<0.20), small (0.20–0.49), medium (0.50–0.79), and large (≥0.80) [20]. Meta-analyses were performed using the random-effects model. The *I*^2^ statistic was used to evaluate heterogeneity. *I*^2^ values were interpreted as low (<50%), moderate (50–75%), and high heterogeneity (>75%). The statistical significance threshold was set at *p* < 0.05. All analyses were performed using the Comprehensive Meta-Analysis software, version 2 (Biostat Inc., Englewood, NJ, USA).

## 3. Results 

### 3.1. Search Results

**Figure 1:**
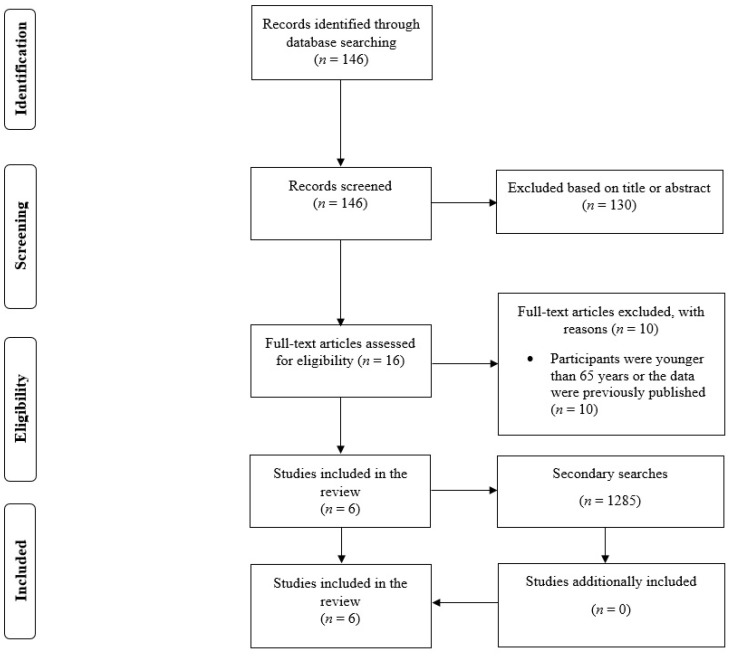
Flow diagram of the search process.

In the primary search that involved examining bibliographic databases, there were 146 results. Out of this pool of references, 130 results were excluded based on the title or abstract. Thus, 16 full-text studies were read. Ten studies were excluded from the review, most commonly because they included participants that were younger than 65 years. Six studies were included in the review [11,12,13,14,15,16]. When examining articles that cited the included studies, there were 1079 results, but no additional studies were included. There were 206 articles in the reference lists; however, this part of the search process also did not result in the inclusion of any additional studies. In summary, six studies were found to satisfy the inclusion criteria (Figure 1) [11,12,13,14,15,16]. While there were only six included studies, there were up to eight groups in the meta-analysis. Specifically, one study [12] presented data separately for older males and females, while another study [14] included two groups that consumed either a recombinant human growth hormone or a placebo. Accordingly, the data for the multiple groups were analyzed separately, as these were independent participants with their individual pre-intervention, post-intervention, and post-detraining data presented.

### 3.2. Summary of Studies

The sample sizes in the included studies ranged from 5 to 19 participants. Three studies included a mixed-sex sample; two studies included only males, while one study included only females (Table 1). The training interventions lasted from 9 to 24 weeks and involved a training frequency of 2 to 3 days per week. The detraining phase lasted between 12 and 52 weeks. All studies evaluated muscle hypertrophy of the quadriceps muscle.

### 3.3. Methodological Quality

Two studies scored 5 points on the PEDro checklist and were classified as being of fair methodological quality (Table 2). Four studies scored 6 or 7 points and were classified as being of good methodological quality.

### 3.4. Meta-Analysis Results

**Figure 2:**
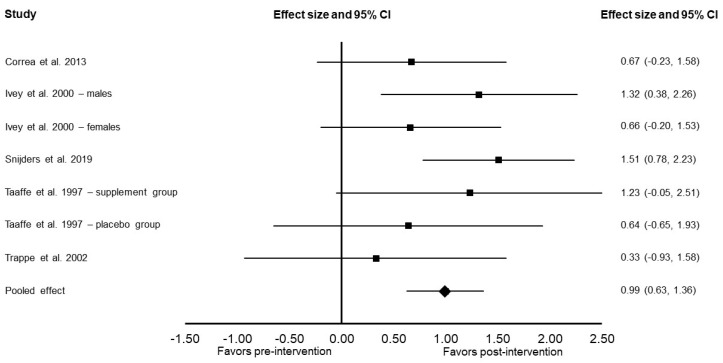
Forest plot presenting the results of the meta-analysis examining the effects of resistance training on muscle size in older adults. Data are reported as Cohen’s *d* (effect size) and 95% confidence interval (CI). The diamond at the bottom presents the overall effect. The plotted squares denote effect sizes, and the whiskers denote their 95% CIs. The statistical weight of the studies ranged from 7.4% to 19.7% [11,12,13,14,16].

Compared to the baseline data, there was a significant increase in muscle size following the resistance training intervention (Cohen’s *d*: 0.99; 95% confidence interval: 0.63, 1.36; *p* < 0.001; *I*^2^ = 0%; Figure 2). Sensitivity analysis did not alter these results.

**Figure 3:**
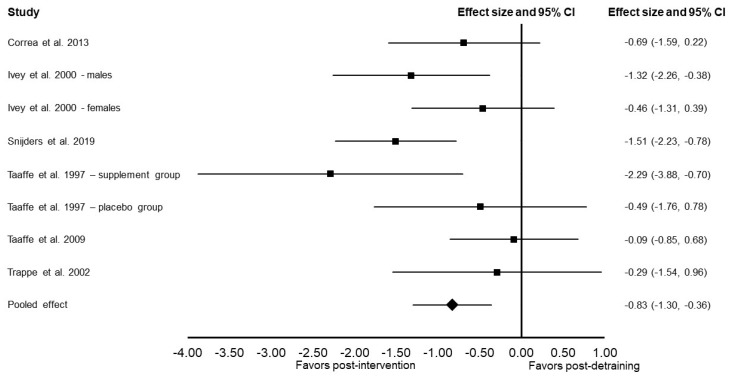
Forest plot presenting the results of the meta-analysis examining the effects of training cessation (detraining) on muscle size in older adults. Data are reported as Cohen’s *d* (effect size) and 95% confidence interval (CI). The diamond at the bottom presents the overall effect. The plotted squares denote effect sizes, and the whiskers denote their 95% CIs. The statistical weight of the studies ranged from 6.7% to 16.1% [11,12,13,14,15,16].

Compared to the post resistance training data, there was a significant decrease in muscle size following training cessation (Cohen’s *d*: −0.83; 95% confidence interval: −1.30, −0.36; *p* = 0.0005; *I*^2^ = 46%; Figure 3). Sensitivity analysis did not alter these results.

In subgroup analyses, there was no significant decrease in muscle size following 12–24 weeks of training cessation (Cohen’s *d*: −0.60; 95% confidence interval: −1.21, 0.01; *p* = 0.06; *I*^2^ = 36%). There was a significant decrease in muscle size following 31–52 weeks of training cessation (Cohen’s *d*: −1.11; 95% confidence interval: −1.75, −0.47; *p* < 0.001; *I*^2^ = 44%).

## 4. Discussion

There are several important findings arising from this review. First, resistance training increases muscle size in older adults. Second, training cessation is associated with a decrease in muscle size. Third, the decrease in muscle size might be related to detraining duration, with greater muscle loss occurring during longer duration detraining periods. From a practical perspective, these results are of importance for older adults who might be unable to participate in resistance training at specific periods due to various reasons (e.g., travel, loss of motivation).

The data presented herein further confirm that resistance training induces muscle hypertrophy in older adults [7]. Previous research established that older adults experience a reduced muscle protein synthetic response to protein intake, a physiological adaptation termed “anabolic resistance” [21]. Due to these physiological effects, it was hypothesized that older adults might also experience an attenuated muscle hypertrophy response. However, the pooled meta-analytical data presented herein indicate that resistance training produced large effects on muscle hypertrophy. Furthermore, a recent meta-analysis focused on very elderly adults (75+ years of age) and observed that resistance training was effective for muscle hypertrophy [22]. Therefore, these data reinforce the positive effect of resistance training on increasing muscle size in older adults [7]. Such effects may occur even with a relatively short time commitment, as the training sessions in one of the included studies lasted around 10 min (3 times per week) and increased quadriceps muscle size [16].

While resistance training increased muscle size, detraining was associated with a decrease in muscle size. Muscle hypertrophy occurs due to several factors, including a positive muscle protein balance and satellite cell addition to muscle fibers [23,24,25]. Resistance exercise increases both muscle protein fractional synthesis rate and satellite cell content [25,26]. Thus, when this stimulus is removed, there will inherently be less daily protein synthesis, which could result in a net loss in protein pool size. Additionally, satellite cell content decreases during detraining, which is likely another physiological contributor to the decrease in muscle size occurring during training cessation [25]. However, data also indicate that the number of satellite cells per fiber remains elevated at 3, 10, and 60, but not 90 days of detraining [25]. Therefore, detraining duration is an important factor that needs to be considered when interpreting the results. Specifically, the included studies varied in their detraining phase duration, as some used 12 weeks, while others used up to a year (Table 1). It seems likely that the negative effect of detraining increases alongside its duration. For example, a large decline in muscle size was observed in one study [13] that used a detraining phase of 1 year (Cohen’s *d*: −1.51), whereas a much smaller decline was observed in a study that used 12 weeks of training cessation (Cohen’s *d*: −0.69) [11]. It might be that muscle size is relatively preserved over shorter detraining periods (e.g., 2 weeks). In support of this idea, a recent study on adolescent athletes reported that three weeks of detraining did not significantly decrease muscle thickness [27]. Indeed, the subgroup analyses indicated that the magnitude of muscle loss increases along with detraining duration. Future studies, however, are needed to establish the time course of muscle size changes during detraining in older adults [28]. Studies may consider using a longer duration detraining period (e.g., 6 months) where muscle size is evaluated periodically (e.g., every 2–4 weeks). Additionally, future studies are also needed to establish the association between resistance training and detraining duration. Specifically, a recent meta-analysis reported that muscular strength gains in older adults are maintained as long as the detraining phase is shorter than the duration of the training intervention [29]. Whether this also applies to muscle hypertrophy remains to be determined.

Only a limited number of meta-analyses explored the effects of resistance training cessation on outcomes related to skeletal muscle [29,30,31]. Bosquet et al. [30] examined the effects of detraining on muscular strength, power, and endurance. This meta-analysis reported that all three muscular qualities were substantially reduced in older adults following detraining (Cohen’s *d*: −0.46 to −0.85). Another meta-analysis explored the effects of detraining on muscular strength in middle-aged and older adults [29]. This meta-analysis also reported a reduction in muscular strength, which was moderated by the duration of resistance training and subsequent detraining and the utilized training loads [29]. Finally, one recent meta-analysis [31] explored the effects of training cessation on the rate of force development (RFD). Unlike adaptations observed for muscular strength, power, and endurance, this analysis found that resistance-training gains in RFD are maintained during detraining [31]. However, this analysis did not focus directly on older adults, as only two out of the seven included studies involved older adults as participants. Thus, the present meta-analysis contributes to the current evidence base, as it is the first to explore detraining′s effects on muscle size in older adults.

There are several limitations of the review that need to be considered when interpreting the results. One limitation is that there were only six included studies. While this number of included studies did not likely influence statistical power (given that significant differences were detected in the analyses), it did prevent additional analysis for potential moderators (e.g., sex). Furthermore, the meta-analytical data are specific to the quadriceps muscle, given that this was the evaluated muscle group in all included studies. Data indicate that quadriceps muscle strength is associated with a risk of falls in older adults, which likely explains why this muscle group was the focus of the included studies [32]. Future studies are needed to explore the effect of detraining on muscle size in other muscle groups in older adults. All six studies included untrained older adults as participants. Therefore, these results may not necessarily apply to older adults who are already resistance-trained. Indeed, previous studies have reported that trained vs. untrained individuals experience divergent responses to detraining [30]. Thus, future research on the population of resistance-trained older adults is needed. Finally, during the detraining phase, studies generally reported that the participants were instructed to resume their normal lifestyle (i.e., no physical exercise involved). It is conceivable that more physically active individuals will experience a smaller loss of muscle size during detraining compared to those who are generally sedentary. Future research may consider examining the effects of detraining on muscle size among individuals with varying habitual physical activity levels.

## 5. Conclusions

This meta-analysis explored the effects of resistance training and detraining on muscle hypertrophy in older adults. While resistance training increased muscle size in older adults, training cessation was associated with a decrease in muscle size. However, the decrease in muscle size might be related to detraining duration, with greater muscle loss occurring during longer duration detraining periods. From a practical perspective, these results are of importance for older adults who might be unable to participate in resistance training at specific periods due to various reasons (e.g., travel, loss of motivation).

## Figures and Tables

**Table 1:** Summary of the included studies.

Study	Participants	Resistance Training	Detraining Duration	Muscle Hypertrophy Assessment
Correa et al. (2013) [11]	10 sedentary elderly women (67 ± 5 years)	12 weeks, 2 times per week; 2–4 sets of 8–20 repetitions using 60–80% of 1 RM	12 weeks	Quadriceps muscle volume using B-mode ultrasound
Ivey et al. (2000) [12]	11 older men and 11 older women (65–75 years)	9 weeks, 3 times per week; 5 sets of 5–20 repetitions using 50–80% of 1 RM	31 weeks	Quadriceps muscle volume using MRI
Snijders et al. (2019) [13]	19 older men and women (65+ years)	24 weeks, 3 times per week; 2–4 sets of 8 repetitions using 80% of 1 RM	1 year	Quadriceps muscle cross-sectional area using CT
Taaffe et al. (1997) [14]	11 older men (65–77 years)	24 weeks, 3 times per week; 3 sets of 8 repetitions using 75% of 1 RM	12 weeks	Biopsies of the vastus lateralis muscle
Taaffe et al. (2009) [15]	13 older men and women (65–83 years)	24 weeks, 2 times per week; 3 sets of 8 repetitions using 65–75% of 1 RM	24 weeks	Quadriceps muscle volume using CT
Trappe et al. (2002) [16]	5 older men (70 ± 4 years)	12 weeks, 3 times per week; 3 sets of 10 repetitions using 80% 1 RM	24 weeks	Quadriceps muscle cross-sectional area using CT

MRI: magnetic resonance imaging; CT: computed tomography; 1 RM: one-repetition maximum.

**Table 2:** Results from the PEDro checklist.

Study	Item 1	Item 2	Item 3	Item 4	Item 5	Item 6	Item 7	Item 8	Item 9	Item 10	Item 11	Score
Correa et al. (2013) [11]	Yes	No	No	Yes	No	No	No	Yes	Yes	Yes	Yes	5
Ivey et al. (2000) [12]	Yes	No	No	Yes	No	Yes	Yes	Yes	Yes	Yes	Yes	7
Snijders et al. (2019) [13]	Yes	No	No	Yes	No	No	No	Yes	Yes	Yes	Yes	5
Taaffe et al. (1997) [14]	Yes	Yes	No	Yes	No	No	No	Yes	Yes	Yes	Yes	6
Taaffe et al. (2009) [15]	Yes	Yes	No	Yes	No	No	No	Yes	Yes	Yes	Yes	6
Trappe et al. (2002) [16]	Yes	No	No	Yes	No	Yes	Yes	Yes	Yes	Yes	Yes	7

No: criterion is not satisfied; Yes: criterion is satisfied.

## Data Availability

Not applicable.
